# Extended Tromograph Surveys for a Full Experimental Characterisation of the San Giorgio Cathedral in Ragusa (Italy)

**DOI:** 10.3390/s23020889

**Published:** 2023-01-12

**Authors:** Giacomo Imposa, Sabrina Grassi, Alberto Barontini, Gabriele Morreale, Salvatore Russo, Paulo B. Lourenço, Sebastiano Imposa

**Affiliations:** 1 Department of Architecture Construction Conservation, University IUAV of Venice, Dorsoduro 2206, 30123 Venice, Italy; 2 Department of Biological, Geological and Environmental Sciences—Earth Science Section, University of Catania, Corso Italia, 57, 95129 Catania, Italy; 3 Department of Civil Engineering, ISISE (Institute for Sustainability and Innovation in Structural Engineering), University of Minho, 4800-058 Guimarães, Portugal

**Keywords:** heritage, HVSR, seismic tomography, MASW, tromograph, ambient vibration testing, operational modal analysis

## Abstract

Geophysical surveys are widely used to reconstruct subsoil seismo-stratigraphic structures with a non-invasive approach. In this study the geophysical surveys were carried out with the aim to characterise the San Giorgio Cathedral in Ragusa (Italy) and the area on which it is built from a dynamic point of view. A 3D subsoil model was realised through the integration of two active (i.e., seismic tomography and multichannel analysis of surface waves) and one passive seismic technique (horizontal to vertical spatial ratio). The instrumentation used for the latter method consists of a tromograph (Tromino^®^), which is also employed for the characterisation of the building, focusing on the façade and the dome, by means of an ambient vibration test, processed through the standard spectral ratio and frequency domain decomposition methods. Integration of the 3D model, showing the distribution of areas with different physicomechanical characteristics, enables identifying anomalies that are likely attributable to the remains of the ancient Byzantine church of San Nicola. Four lower modes mainly involving the two investigated macroelements are identified. The experimental results outline the advantages of the use of the tromograph both for soil and structural characterisation, especially for massive masonry buildings located in areas with high seismic hazard.

## 1. Introduction

Implementing a holistic and integrated multidisciplinary and multi-methodological investigation strategy is crucial for the preventive conservation of architectural heritage, especially considering the numerous natural and man-made threats to which it is subjected [1]. In this regard, non-destructive diagnostic techniques play a key role in providing a complete overview of the heritage site, including not only the structure but also the subsoil [2]. Indeed, the importance of the analysis of the soil behaviour to the assessment of the whole built heritage system should not be neglected [3]. Nowadays, geophysical survey techniques are widely used as a strategic tool to characterise the soil and identify and investigate underground evidence, as for instance archaeological remains or even structural elements of the building [4,5,6,7,8,9,10]. The widespread use of these methods of investigation is linked to their non-invasive character, their aptitude to be applied to different contexts, especially in urban areas and, in some applications, and the possibility of exploiting the ambient noise originated by natural (e.g., micro tremors, marine waves, wind, meteorological conditions) or anthropic (e.g., human activities, traffic, industrial machinery) sources of vibration [11]. Ambient vibration testing has also been explored for the characterisation of the structural behaviour of historical buildings (e.g., ancient churches), allowing the estimation of their modal properties, namely resonant frequencies, mode shapes, and damping ratios [12,13,14,15], for the assessment of the evolution over time of damage and deterioration, including under extreme hazardous events [16,17,18] and for the calibration of finite element model to perform static and dynamic non-linear analyses [19]. Additionally, it should be noted that the amount of experimental data in the literature are also used to derive empirical formulas for estimating frequencies [20,21].

The present paper aims at testing on a relevant heritage site a promising approach for a cost-effective, less expensive and time-consuming, global characterisation of massive monumental masonry buildings under low-amplitude vibration, which resorts to only one light-weight wireless device, namely a tromograph [16,17] (commercially known as Tromino^®^), used according to the specific requirements of the soil and structural investigation. Tromino^®^ can sample ambient noise with 6 velocimeter channels (high and low resolution) and 3 accelerometer channels, in a frequency range from 0.1 to 1024 Hz [20]. Such devices are portable and wireless; they do not need any fixing system to couple with the structure and above all they allow synchronous data acquisition via radio linking [21], therefore they are perfectly suitable for an expedited non-invasive investigation on site.

San Giorgio Cathedral in Ragusa Ibla (Italy), a UNESCO World Heritage site described in Section 2, is considered a relevant example for the application of this approach, due to its high artistic value and the elevated seismicity of the area where it is located. In Section 3 the geophysical campaign consisting of the integration of three different techniques is illustrated: two active, namely the Seismic tomography and the Multichannel analysis of surface waves (MASW) performed by geophones; one passive, namely the horizontal to vertical spatial ratio (HVSR), performed by tromographs. The latter instrumentation is also adopted to carry out a rapid preliminary dynamic identification of the church, focusing on the façade and on the dome, as further described in Section 4. Finally, in Section 5, conclusions are drawn and future perspectives outlined.

## 2. Description of the Case Study

### 2.1. Geological and Seismological Framework

**Figure 1:**
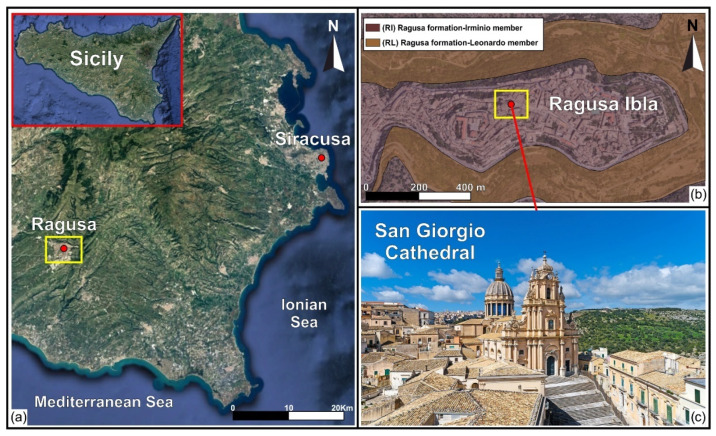
(**a**) Location of Ragusa Municipality (south-eastern Sicily); (**b**) geological map of Ragusa Ibla: RI Ragusa formation—Irminio member (white-grey calcarenites and calcirudites), RL Ragusa formation—Leonardo member (calcisiltites, marls and marly limestones); (**c**) External view of San Giorgio Cathedral.

The Cathedral of San Giorgio of Ragusa Ibla is located in a high seismic hazard zone in the south-eastern Sicily (Figure 1a), commonly known as the Hyblean Plateau. From a geological point of view, Ragusa Ibla is situated on an elongated and almost flat calcarenites hill, 400 m above the sea level and is bordered to the north and south by two river incisions oriented approximately E–W, caused by two tributaries of the Irminio River. The stratigraphic succession of the study area is represented by a carbonate formation, composed of layers of calcarenites and white-greyish marls, called the Ragusa formation (Figure 1b).

**Figure 2:**
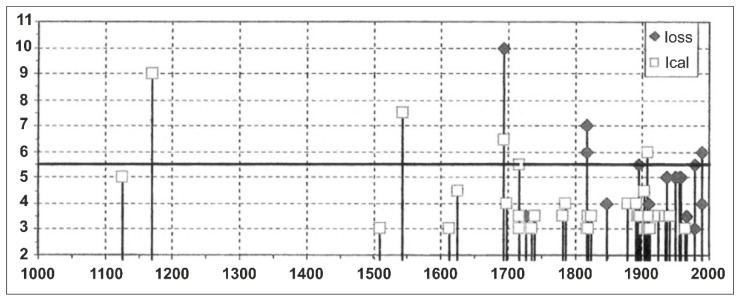
Seismicity of Ragusa over the last millennium, referring to Modified Mercalli Intensity Scale (MCS) Intensity values *I* observed *I_oss_* and the calculated ones *I_cal_*.

The site seismic history shows that it has been affected over the last millennium by many seismic events [22] (Figure 2). The average modified Mercalli intensity scale (MCS) value *I* is equal to about 5–6, based on the observed (*I_oss_* in Figure 2) and the calculated values (*I_cal_* in Figure 2), for which the estimation is based on the method reported in [23]. One of the most destructive earthquakes in Italian history occurred in 1693 [24] and had its epicentre at Val di Noto (Mw 7.3). This earthquake struck Ragusa with an intensity of IX-X on the MCS, destroying most of the ancient masonry buildings, including ecclesiastical and monumental structures, due to their high vulnerability, as well as to the severe ground shaking.

Moreover, other two destructive events hit Ragusa in 1818 and 1990, with epicentre in the Etna region (Mw 5.51) and Augusta (Mw 5.64), respectively. Even though they are recognised as moderate earthquakes, they caused considerable damages, including to modern reinforced concrete buildings [25].

**Figure 3:**
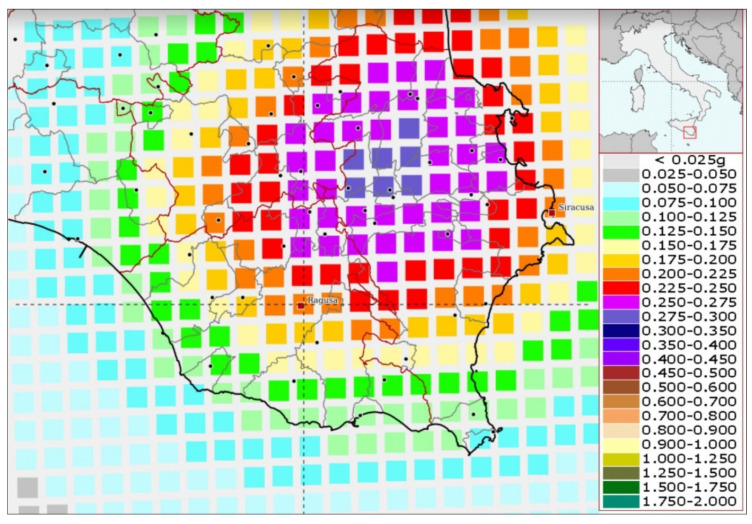
Interactive seismic hazard maps of the city of Ragusa with 10% probability of exceedance in 50 years (return period of 475 years), PGA = 0.200–0.225 g.

**Figure 4:**
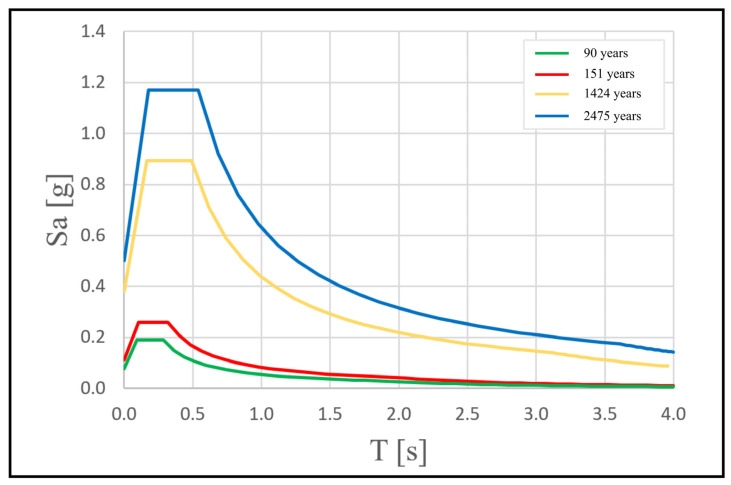
Spectra of the horizontal seismic action provided by Italian building code for different return periods with a nominal life of 100 years and use coefficient 1.5 related with the selected typology of building (e.g., Church).

In Figure 3, the probabilistic assessment of the seismic hazard in the area of Ragusa is shown in terms of peak ground acceleration (PGA) with a 10% probability of exceedance in 50 years, as prescribed by the procedure for the “*Seismic Hazard Assessment for the Italian Building Code*” [26]. For the investigated area, the expected PGA is in the range 0.2–0.225 g, with the uncertainty on the prediction equal to 0.025 g. In Figure 4., the acceleration response spectra of the horizontal seismic action are presented, according to the Italian building code, considering a rigid soil (A class, Vs30 > 800 m/s) and level ground. They are associated to the four limit states related to their performance (operative condition, damage limitation, significant damage, and near collapse) expressed in terms of return periods for the studied building with use coefficient equal to 1.5 and rated life about 100 years.

### 2.2. Historical and Architectural Background

**Figure 5:**
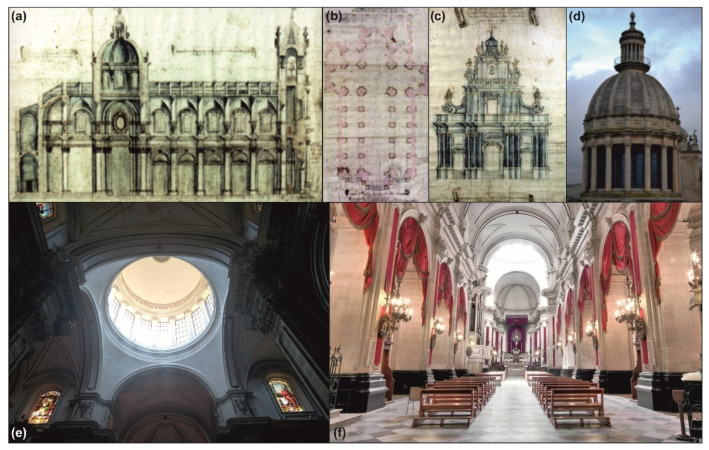
Gagliardi’s original drawings of nave (**a**), general layout (**b**), façade (**c**); view of the current dome from external (**d**) and internal perspective (**e**), view of the main nave (**f**).

The project of the cathedral was commissioned in 1744 and awarded to Rosario Gagliardi, one of the greatest exponents of the Sicilian Baroque. The aim was to dedicate a new church to the patron saint of the city, which was partially destroyed during the “Val di Noto” earthquake in 1693. The new church is located in the city centre, occupying the area of the ruined Byzantine church of San Nicola. The Syracusan architect was an eyewitness to the destructive effect of the catastrophe on the architecture of his homeland. Based on this experience, he paid particular attention to the possible effect of an earthquake on the architectural features and structural elements of the church. Among them it is worth mentioning the most significant components of his well-known style, influenced by Borromini and Bernini, such as the massive pyramidal façade with the built-in bell tower, the magnificent neo-classical dome, and the impressive staircase that serves as a platform for the façade (Figure 5). The raised location of the church and the divergent perspective axis between the dome and the façade lend dynamism to the entire building.

The layout of the cathedral consists of three aisles divided by two rows of ten pillars each, with a transept and a semi-circular apse, following the typical Latin cross scheme (Figure 5a,b). In addition, there are thirteen chapels along the lateral naves adorned with precious frescoes. The massive structure is 68 m long and 27 m wide, with the highest point placed on the dome at a height of 42 m. In the façade, a long spiral staircase on the left-hand side follows the incorporated bell tower, ending at the top with a bulb cusp according to the traditional Capuchin style. Three orders of columns separate the façade in three sections, with the central one being slightly convex, and the pyramidal shape determines a gradual lightening toward the top of the element. Despite the latter, as widely shown in the recent experience of “L’Aquila” earthquake in 2009, these types of curved facades have a high vulnerability that can trigger collapse mechanisms as in the cases of “Anime Sante” Church [27] and “Immacolata Concezione” Church in Paganica [28].

The actual dome, the magnificent character-defining feature of the building (Figure 5), is the result of a reconstruction in 1820 carried out by Carmelo Cultraro, after the collapse of the original one, due to the weakening caused by the “Etna” earthquake. Cultraro drew inspiration from an illustration of the Pantheon in Paris, and built the current version twice the size of the previous dome. The dome is a double shell, with the interior made of masonry and the exterior of stone. It has a diameter of almost 12 m and is supported by 16 twin Corinthian columns resting on the 4 octagonal pillars located at the intersection between naves and transept. The dome culminates with a lantern on the top characterised by its distinctive blue stained-glass. In the 2000s, upon the sudden partial collapse of the nearby Cathedral of Noto [29], the Superintendence commissioned the strengthening of the macroelement by means of steel bars inside each column and a reinforced concrete curb at the base of the drum.

In 1989, archaeological investigations were carried out in a small portion near the apse of the Cathedral of San Giorgio and the area surrounding the church. The cutting of the rock slope for the foundations of the apse was identified, together with a small wall portion of a staircase likely belonging to the San Nicola church and a soil deposit between the apsidal wall of the cathedral and the remains of the previous structure.

## 3. 3D Subsoil Modelling by Geophysical Surveys

An extensive geophysical field survey was carried out to define the physical-mechanical properties of the subsoil on which the San Giorgio Cathedral is built and to identify the possible presence of archaeological remains and/or crypts belonging to the ancient Church of San Nicola. Two different geophysical methods were applied: passive seismic survey HVSR (horizontal to vertical spectral ratio) and active seismic techniques (seismic tomographies and MASW, multichannel analysis of surface waves). Furthermore, an integrated analysis of the obtained results was carried out in order to verify the validity of the identified anomalies.

### 3.1. Methods and Field Surveys

The seismic refraction is an active seismic survey based on the study of elastic waves propagation. There is a link between the seismic waves’ velocity and the physicomechanical features of the lithotypes; the higher the seismic waves velocity, the better the subsurface characteristics.

**Figure 6:**
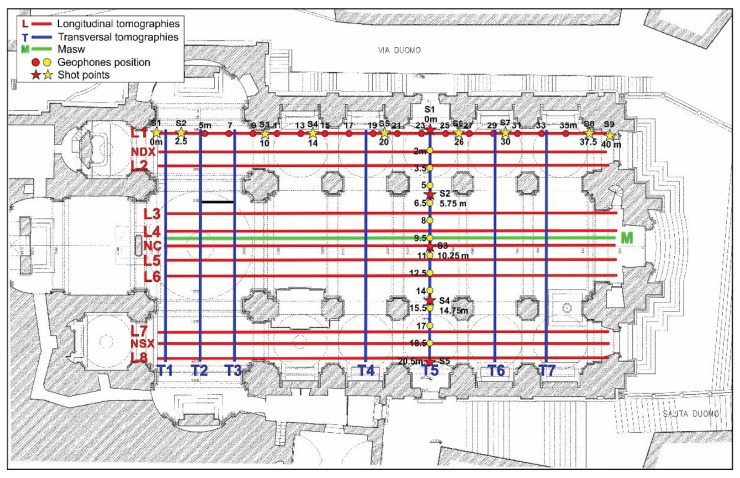
Seismic tomographies and MASW acquisition schemes.

This geophysical method renders a two-dimensional section of seismic waves velocity distribution in the subsurface, allowing the identification of any discontinuities, cavities/tombs [30,31]. Inside the cathedral, 18 seismic refraction tomographies were performed, in two main directions, 11 in the longitudinal direction along the naves and 7 orthogonal to the previous ones, according to the distribution shown in Figure 6.

A 40-m-long array, consisting of 16 geophones with 4.5 Hz frequency spaced 2 m apart, plus one geophone used as a trigger, was used for longitudinal tomographies. An 8 kg sledgehammer beating on a wooden base coupled tightly to the flooring was used as a source of energization.

For the longitudinal lines, the distance of external shot points was 5 m, where the internal shots were distributed in this direction according to the scheme reported in Figure 6. For the tomographies orthogonal to the naves, we used a 20.5 m array, consisting of 12 geophones spaced 1.5 m apart, plus one geophone as a trigger. The distance of external shot points for these lines was 2 m, where the internal shots were distributed according to the scheme shown in Figure 6.

To define the 1D shear wave velocity profile of the subsoil below the church, a MASW survey was performed. MASW is an active seismic method based on the sampling and analysis of Rayleigh wave’s dispersion [32,33].

The MASW survey was performed along the central nave using a digital multichannel array, consisting of 16 vertical geophones, with a natural frequency of 4.5 Hz, spaced 2 m apart for a total coverage of 30 m (Figure 6). Four shots were performed at 5 m from the first and last geophone, using the stacking method (improve the signal to noise ratio). For each shot, the signal was recorded for 2 s with a sampling frequency of 512 Hz.

In the same area studied with the active seismic surveys, an extensive passive seismic single-station field survey was performed using the HVSR technique [34,35]. This method is based on the ambient noise sampling [36]. Recording the ground motion in the three-space components, it is possible to estimate the site response and the resonance frequency [37] by calculating the spectral ratio between the average of horizontal components on the vertical one.

**Figure 7:**
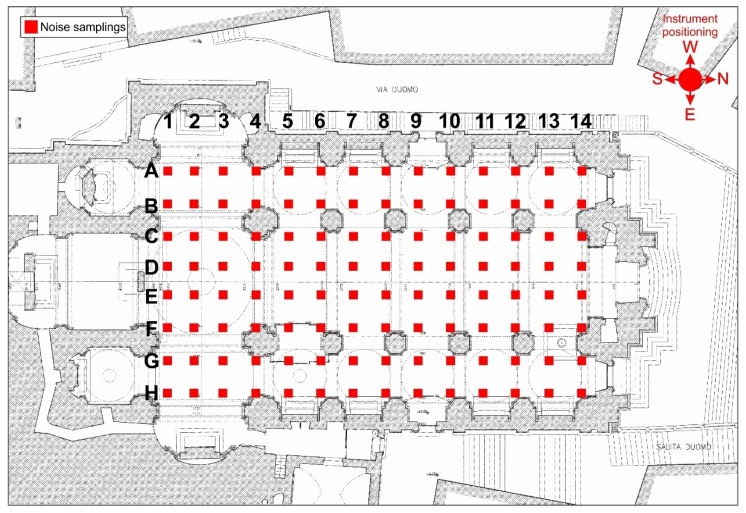
Locations of ambient noise samplings.

The passive seismic single-station recordings were carried out on the flooring with a distance between the sampling points of 3 m, at the nodes of a specially designed grid (Figure 7). The ambient noise measurements were performed through n.6 digital three-component velocimeters, known as Tromino^®^, using a sampling frequency of 512 Hz, for 16 min. For all measurements, the N–S instrumental axis was oriented according to the longitudinal axis of the cathedral. The 112 samplings performed were carried out on the same day.

### 3.2. Data Processing

**Figure 8:**
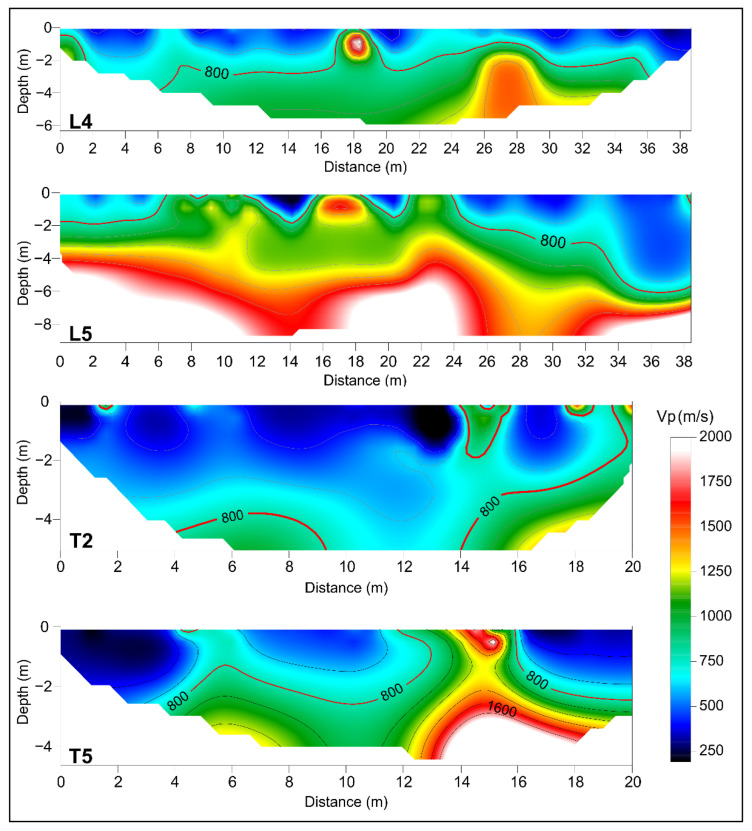
Examples of longitudinal (L) and transversal (T) seismic tomographies. The red line highlights the isoline related to V_p_ = 800 m/s.

The seismic refraction data processing was performed using the SeisOptim software based on generalised simulated annealing optimization method (GSAO) [38]; this algorithm allows a non-linear inversion procedure of the recorded seismic waves’ arrival times. Longitudinal and transversal seismic tomography sections show a similar trend. The sections highlight the presence of portions characterised by low velocity values of compressive waves, Vp ≈ 400–600 m/s (Figure 8), from ground level to a depth of about 4 m, attributable to material with poor physicomechanical properties. In addition, the different sections show the presence of portions characterised by higher compression wave velocity values than the previous ones, Vp > 1000–1200 m/s (Figure 8); these values can be associated with an improvement in subsoil physical-mechanical properties.

**Figure 9:**
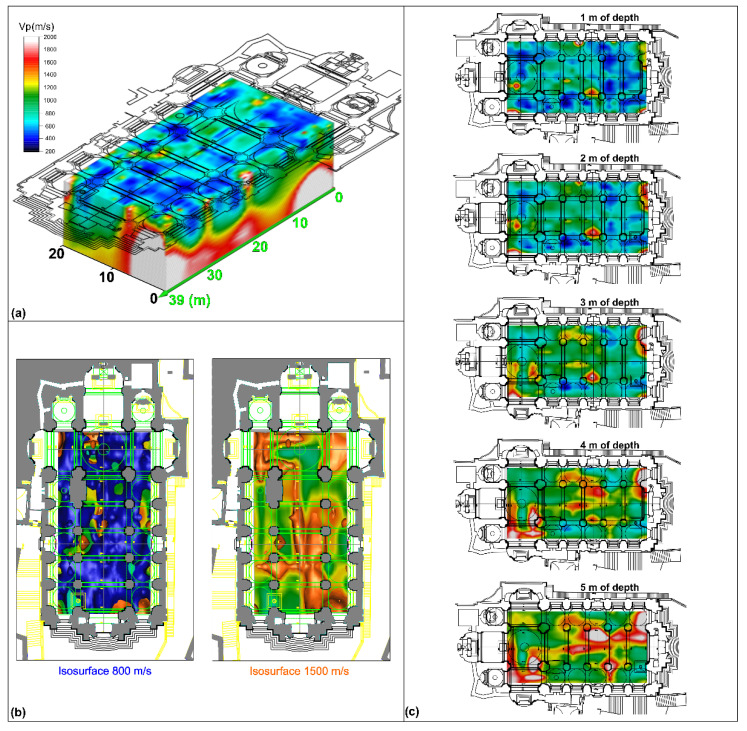
(**a**) 3D subsoil model obtained from the interpolation between seismic tomography sections; (**b**) isosurfaces for longitudinal wave velocity values (V_p_) of 800 and 1500 m/s; (**c**) depth slices.

Successively, an interpolation between the 2D seismic tomography sections was performed using a contouring function to reconstruct a 3D seismic stratigraphic subsoil model, showing the distribution of longitudinal wave velocity values in the three dimensions (Figure 9a). From the 3D model, the isosurfaces showing different wave velocity values were extracted. The isosurface characterised by velocity values of about 800 m/s was highlighted (Figure 9b), which represents the base of the low-velocity areas probably linked with the presence of filling material. The isosurface with velocity values of 1500 m/s (Figure 9b), was also highlighted, which could be linked to the lithotypes characterising the subsoil of the investigated site (i.e., calcarenites belonging to the Ragusa Formation).

In addition, several depth slices with a step of 1 m were extracted from the 3D model, which made it possible to observe the extension in depth of low-velocity areas and detect the distribution of the high-velocity zones (Figure 9c). Depth slices show that the low-velocity areas extend down to a depth of about 4 m. Below this depth, anomalies characterised by low-velocity values tend to decrease, while the presence of areas with high-velocity values begins to become more widespread.

The ambient noise samplings were processed by dividing the traces into windows of 20 s each, using a triangular function and 10% smoothing in the frequency range 0.5–256 Hz (i.e., half sampling frequency). The reliability of the curve obtained and the clarity of the peaks are determined based on the criteria expressed in the SESAME guidelines [39]. This involves assessing the reliability of the results. The maximums of the spectra provide the value of the resonance frequency. Furthermore, the amplitude of the maxima is directly proportional, but not linearly, to the magnitude of the impedance contrast. The impedance contrast reflects the variation of shear wave velocity and density in the subsurface.

**Figure 10:**
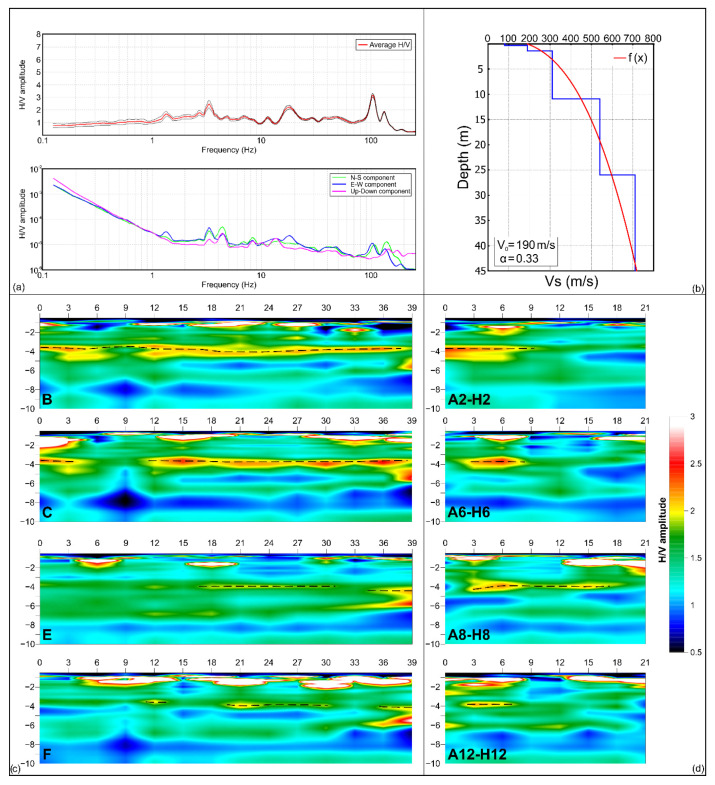
(**a**) Example of H/V spectrum obtained from ambient noise processing and relative Fourier spectral components graph; (**b**) Vs-depth profile obtained by MASW data processing, the red curve is the function that fits the data characterised by minimum misfit with velocity profile; examples of longitudinal (**c**) and transversal (**d**) impedance contrast sections (see Figure 4 for location). The dotted lines highlight an impedance contrast observed in all the sections.

All the H/V spectra show a similar average pattern (Figure 6a). In almost all spectra, the presence of a peak at higher frequencies, defined as significant because it is characterised by H/V amplitude > 2, is attributable to a possible stratigraphic variation, according to the trend of the single components Fourier spectra. In fact, when an impedance contrast is present in the subsoil, the spectra of single components display a typical “eye shape” formed between the horizontal and vertical components [40]. Observing the spectra (Figure 10a), it is possible to note the presence of a broadband frequency peak between 1 and 10 Hz, characterised by H/V amplitude values less than two, which while not significant [39] is nevertheless indicative of the possibility of amplification within that frequency range in the event of an earthquake.

Through the inversion of Rayleigh waves acquired with the MASW survey [41,42] an estimate of near-surface shear-wave velocity was obtained. The Vs-depth profile (Figure 10b) shows a shallow layer, between 0–2.5 m of depth, characterised by a value of shear waves of about 90 m/s. Below this layer, the shear waves velocity values increase with the depth, reaching a maximum of 700 m/s. The first layer, characterised by low velocity values, is associated with the presence of fill material, while the significant increase of the velocity values in the underlying layers, in accordance with the geological features of the site, is attributable to the presence of more compact lithotypes such as calcarenites belonging to the Ragusa formation.

By integrating the data of the H/V spectra with the information on the shear waves velocity distribution, it was possible to reconstruct 2D seismic stratigraphic sections known as impedance contrast sections. These sections show the distribution of H/V spectral ratio amplitude values in the subsoil, allowing identifying any horizontal and vertical variations [43,44,45]. Impedance contrasts are determined by the variation of seismic impedance between different layers defined as the product between the density of the medium and the velocity of the shear waves. This parameter affects the propagation of seismic energy content between layers with different physicomechanical properties.

**Figure 11:**
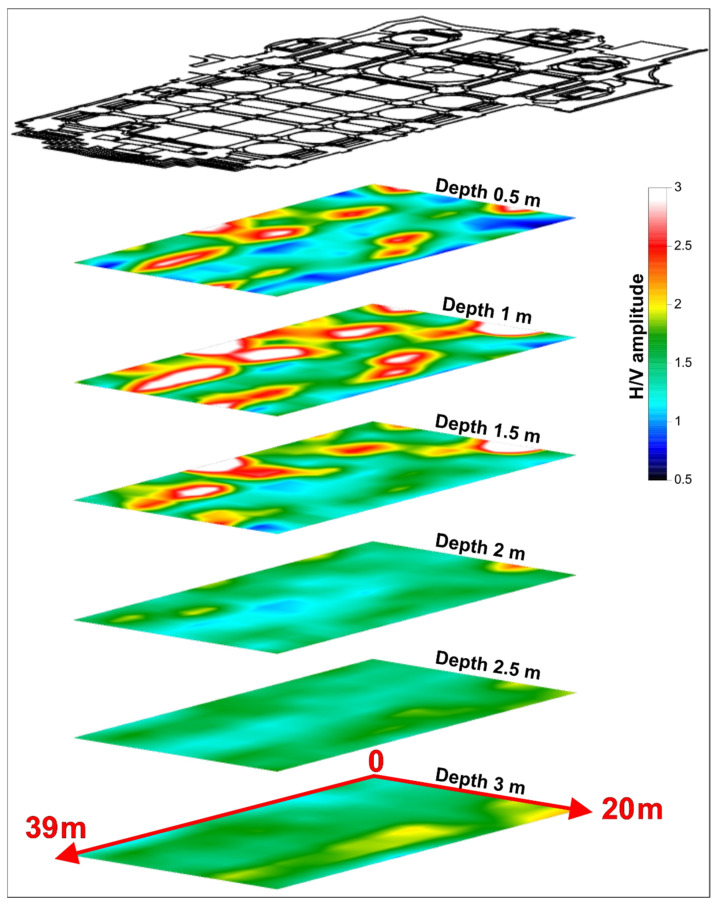
Depth slices showing the variation of the H/V spectral ratio amplitude with the depth.

The shallow wave velocity V_0_ and the proportionality coefficient between depth and velocity α, obtained by fitting the 1D Vs-depth profile (Figure 10b) were used in the Ibs von Seht and Wohlenberg equation [46] to convert H/V spectra frequency values into depth values. This allowed plotting the spectral ratio amplitude values in depth. By recording ambient noise at the nodes of a regular grid (Figure 7), it was possible to interpolate the H/V spectra to obtain 8 longitudinal and 14 transversal impedance contrast sections. All sections are characterised by an approximately 0.50 m thick shallow layer that shows H/V amplitude < 1, which can be associated with the effect of the church flooring standing on the fill material used to level the surface (Figure 10c,d). Down to 2 m depth, the sections show portions characterised by H/V amplitude values < 1 interposed to areas characterised by high H/V amplitude values > 2 (Figure 10c,d). Below this layer, the presence of impedance contrasts is observed down to a depth of approximately 4 m. The areas characterised by these impedance contrasts present H/V amplitude values > 2 and reveal the transition between materials characterised by different physical-mechanical features. Furthermore, all sections show the presence of a continuous layer characterised by H/V amplitude < 1 between 6 and 8 m depth (Figure 10c,d). Successively, an interpolation between the impedance contrast sections was performed to reconstruct a 3D subsoil model. From this model, depth slices spaced 0.50 m apart were extracted to observe the depth trend of the H/V spectral amplitude (Figure 11).

Analysing the depth slices reveals the evident presence of many areas characterised by values of H/V spectral amplitude > 2 interposed with H/V spectral amplitude < 1 areas in the depth range 0.5–1.5 m. Below 2 m of depth, the slices, related to this processing, do not show the presence of any particularly significant anomalies. For this reason, it was not deemed necessary to show slices related to depths greater than 3 m.

### 3.3. Geophysical Integrated Approach

A mathematical integration approach was used to obtain a quantitative evaluation of the data, which involved the sum and product between the data obtained from active seismic (seismic tomographies) and passive seismic surveys [47]. Some assumptions are necessary in order to apply such approach. We denote the number of investigation techniques performed with the letter *M*. It is assumed that the result of the investigation denoted as m_i_ with i = 1,…, *M*, is a function *f_mi_* (r), that represent the anomaly detected indicator with the different geophysical methods, where *r* is a 3-dimensional position vector. Finally, the probable depth is defined as z_m_ at which the archaeological target is expected to be found analysing the behaviour of the function *F_mi_* (r) with *f_mi_* (*x*, *y*) the restriction of *F_mi_* (r) on the plane *z* = z_m_,

Data functions *F_mi_* (*x*, *y*) were compared numerically with each other to perform a quantitative integration of geophysical methodologies applied. It is necessary to work with a-dimensional values obtained from experimental data. Thus, it is possible to represent the normalised function *F_mi_* (*x*, *y*) as an indicator of the anomaly linked to archaeological targets. This function has values equal to 1 at the points where there is the greatest discrepancy between the data function and the undisturbed value. We are therefore in a position to quantitatively integrate the results obtained by averaging the functions that operate as indicators of anomalies, by means of the following equation (Equation (1)):

(1) $$\bar{F}\;\left(x,y\right)\;\frac{1}{M}\overset{M}{\underset{m_{i}}{\sum }}F_{mi}\left(x,y\right)$$

This type of approach makes it possible to obtain information on each of the applied methods, and in particular, it is possible to define the distribution of anomalies detected by at least one of the methods. In fact, this function has values of 0 where all methods do not show anomalies and values of about 1 where the results show a maximum anomaly pattern. Further, by performing the product (Equation (2)) between the geophysical data, it is possible to highlight the areas where all methodologies showed the presence of anomalies:

(2) $$F^{*}\left(x,y\right)=\overset{M}{\underset{mi}{\prod }}F_{mi}\left(x,y\right)$$

**Figure 12:**
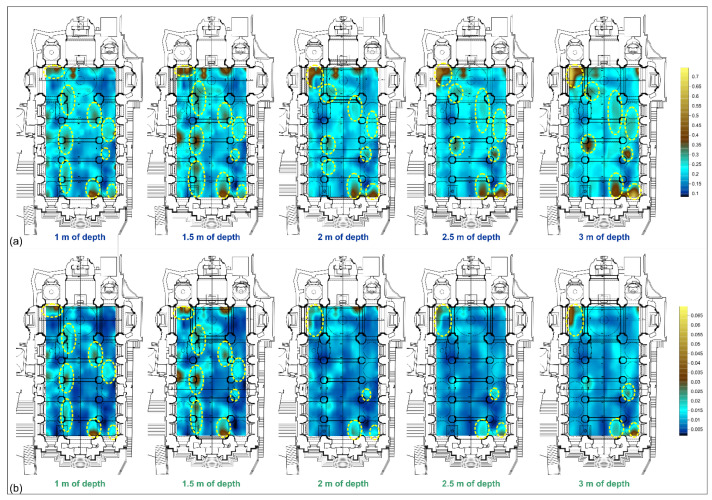
Maps of anomalies between 1 and 3 m of depth obtained from the sum (**a**) and product (**b**) between passive seismic and seismic tomography data. The yellow dashed circles highlighting the anomalies identified in the maps.

The results of the mathematical integration approach between seismic tomography and HVSR data are shown in Figure 12. Data for the anomalies detected between 1 and 3 m of depth with the different methodologies extracted from the respective 3D models, using a step of 0.5 m. Then, as provided by the integration approach proposed by Piro and Gabrielli, 2009 [48], the data were normalised and subsequently mathematically integrated by applying the sum and product approach.

The sum provides a new dataset within which all anomaly information for all methods considered converges, i.e., all data combine to provide a new map. Through this approach, anomalies are highlighted even where one of the methods was not able to reveal sources of anomalies. Figure 12a shows the presence of three aligned anomalies located near the columns of the left aisle; these anomalies are clearly visible down to 2 m deep; below this depth, the anomaly located next to the first column is no more evident. Another two anomalies are observable in the right portion of the central nave near the entrance and between the fourth and fifth columns, respectively. In the right aisle, near the second column and between the third and fourth columns, two anomalies are detectable which become more evident with increasing depth. In addition, close to the right entrance of the church and the left altar, two very pronounced anomalies can be observed in all depth slices (Figure 12a). The use of the product between the results obtained by the different geophysical methods provides a map in which there is an actual correspondence between the anomalies highlighted by both surveys. The map resulting from this process, Figure 12b, very clearly shows the presence of numerous anomalies identified by both methodologies. The presence of the aligned anomalies near the columns of the left aisle is confirmed, in this case they are clearly observable down to 1.5 m of depth. The anomalies detected in the right portion of the central nave appear more circumscribed. The targets identified close to the right entrance and the altars appear less pronounced and extended. Slight anomalies are also observed in the right aisle, as previously identified (Figure 12b).

## 4. Expedited Dynamic Identification

During the extensive geophysical field survey, a preliminary dynamic identification campaign was also carried out to collect relevant information on the natural frequencies and the related mode shapes of the San Giorgio Cathedral, in view of a thorough investigation of the building to be designed and implemented. Indeed, due to time and accessibility constraints, at this stage of the work, it was not possible to extend the study to the entire church. Therefore, a detailed ambient vibration test addressed the façade only. After the analysis of the façade, it was possible to perform an additional test in the dome, although encompassing a single setup. It is worth noting that these two macroelements are particularly vulnerable against the seismic action, due to their features, thus their characterisation is an essential step towards the conservation of the church and a mitigation of the detrimental effects of the earthquake.

### 4.1. Methods and Field Surveys

For the dynamic identification of the structure, the instrumentation used is the same as those of the passive seismic single station surveys. In this case, the identification is first carried out by means of the standard spectral ratio (SSR) method. This consists in computing the ratio of the Fourier spectrum of records in the horizontal directions, measured at vertically aligned points, with a referenced one at the ground level [49]. The natural frequencies of the building are then identified by a simple peak-picking strategy. In particular, the Fourier spectrum is calculated by dividing the signal into non-overlapping windows of 20 s. Each window is detrended, tapered, padded, fast Fourier transformed, and smoothed with triangular windows with a width equal to 1% of the central frequency.

A more robust approach, namely the frequency domain decomposition (FDD) [50], is exploited, to compare and validate the estimated frequencies as well as to extract the mode shapes, by processing more synchronous acquisitions at different measurement points. The comparison between the two methodologies, both suitable for operational modal analysis and operating in frequency domain, stems from a particularity in the peak picking technique for modal identification. In SSR, the peak picking is operated on the distinct individual power spectral densities (PSDs) normalised to the PSD of a reference signal at the ground. The FDD method, instead, aggregates all acquisitions allowing a more robust outcome including the estimation of the mode shapes.

**Figure 13:**
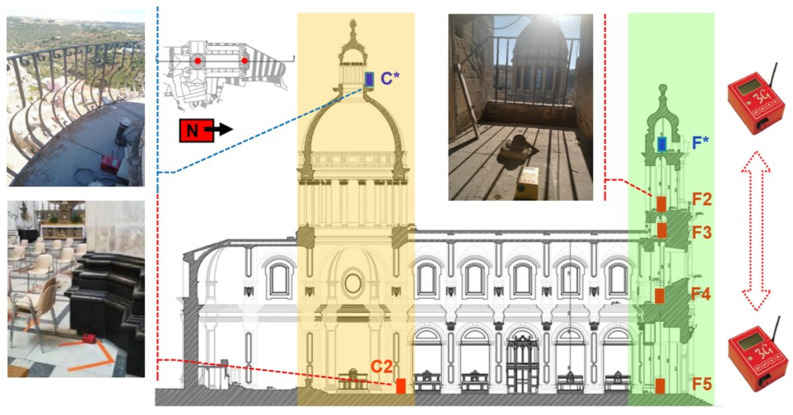
Monitoring scheme of the dome (yellow) and façade (green) and location of the Master (blue) and Rover (red) in the dome (C*–C2) and in the four setups carried out in the façade (F*–F2, F3, F4, F5).

For the acquisitions two devices are employed, one at a fixed, reference location and one roving to measure more points. The built-in radio linking is used to connect them. The selected set-up to obtain the modal parameters of the structure consists of two devices, one at a fixed, reference location and one roving to measure more points. The built-in radio linking is used to connect them. In Figure 13 the monitoring scheme of the façade is represented. Within the façade, there exist five inspectionable levels connected by a spiral staircase, from the ground to the bulb cusp at the top. Due to accessibility limitations and availability of space, the sensors are located in a central position on the right-hand side of the staircase in order to create a vertical alignment, as required by the monitoring protocol. Thus, the so-called Master, F* (in blue), is placed on the top at the height of 30 m, whereas the so-called Rover (in red) assumes a different position in each setup, namely F2, F3, F4 and F5 at 22 m, 19 m, 11 m and ground floor, respectively. In total, four setups are carried out, summing up to five measurement points and 15 degrees of freedom recorded.

Initially the instruments are placed close together so that the radio link is established, and then the Rover is moved to the point of interest, creating a vertical alignment. In each setup, as illustrated in Figure 13, the transducers are placed along the same orientation (i.e., instrumental main axis, namely N–S component, parallel to the longitudinal direction of the building). The acquisition length is approximately 9 min with a sampling rate of 128 Hz. The total duration of the test, including the initial synchronisation, each individual acquisition and the time to move the roving sensor, is about 1 h, demonstrating the versatility and the advantages of these devices which allow expedited and cost-effective testing.

As already mentioned, time and accessibility constraints allow the investigation of the dome through a single setup, and a synchronisation with the recordings in the façade is not possible. The only possible location of the Master, C*, to characterise the macro element, is on the top of the dome, at the height of 40 m and the Rover, C2, at ground level (Figure 13).

### 4.2. Experimental Results

**Figure 14:**
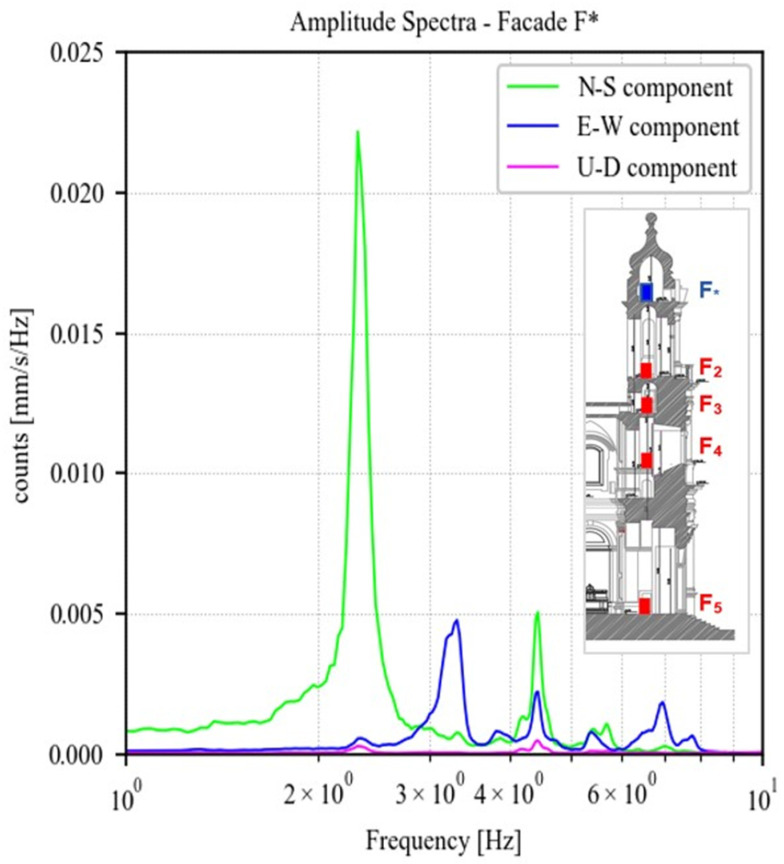
Amplitude of the fast Fourier spectra regarding point F* on the top of the Façade for each component (North–South, East–West, Up–Down).

To identify the main peaks for each component, the amplitude of the Fourier spectra at the highest point of the façade, F*, is shown in Figure 14. A first large amplitude peak emerges at 2.34 Hz in the longitudinal direction (N–S component). A second one, this time in transversal direction (E–W component), is at 3.34 Hz with a lower amplitude. The third peak has a frequency value about 4.45 Hz and affects both the horizontal directions with a higher component along the longitudinal axis of the building.

The comparison between the estimation of the natural frequencies with the SSR and the FDD, reported in Table 1, shows a rather good agreement, demonstrating the viability of the SSR for an expedited processing of the data.

**Figure 15:**
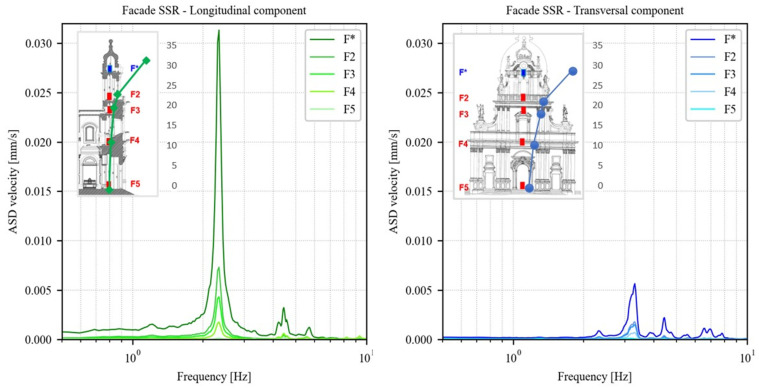
SSR analysis of the façade in longitudinal and transversal direction at Master F* and Rovers F2, F3, F4, F5 locations, with identification of modes f1 at 2.34 Hz (above) and f2 at 3.34 Hz (below), and the related mode shapes obtained by FDD.

The mode shapes of the two first vibration modes, estimated through the FDD, are shown in Figure 15, together with the SSR curves. The first mode consists of an out-of-plane bending of the façade, mainly involving the tympanum, namely from point F3 up to the top on point F*, due to the connection between the façade and the naves. Similarly, in the second mode, the maximum modal displacement is at the tympanum, although this is likely the first global transversal mode of the church.

**Figure 16:**
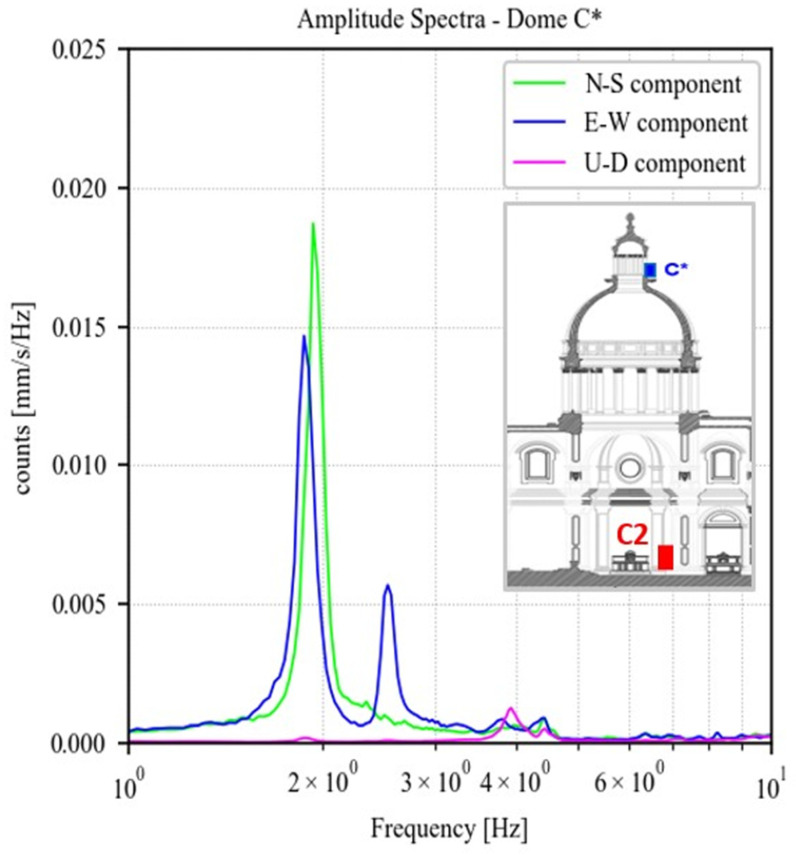
Amplitude of fast Fourier spectra regarding point C* on the top of the dome, for each component (North–South, East–West, Up–Down).

Regarding the dome, Figure 16 shows the amplitude of the Fourier spectra of the signals recorded in the three directions by the Master C* located at the top of the macroelement. Two very closed-spaced peaks emerge at 1.86 Hz and 1.90 Hz, respectively. Although the characterisation of the mode shape requires more measurement points, these are likely two local bending modes of the dome along two main directions, first transversal and then longitudinal, with very similar resonant frequencies due to the symmetry of geometry and boundary conditions. It is worth noting that the global transversal mode estimated for the façade at 3.34 Hz is not identified in this acquisition, thus, the measurement point at the dome may be located close to a node of the mode shape, experiencing a negligible modal displacement.

Finally, the periods of the identified modes for the two macroelements are reported in Table 2. Recalling the response spectra, shown in Figure 4, it is worth noting that the periods, especially for the façade, fall in the range where the spectral acceleration is maximum, meaning that this vulnerable element is expected to undergo a large seismic input.

## 5. Conclusions

A full experimental characterisation of the San Giorgio Cathedral in Ragusa (Italy) is proposed as a consistent application of a rapid but effective dual use of the tromograph. The goal of this preliminary campaign was to assess whether the tromograph is suitable for the characterisation of the modal properties of massive historical structures, under a low amplitude ambient vibration, thus, ensuring the characterisation of the building and its subsoil with a single device.

Passive seismic single-station surveys, processed using the HVSR technique, allowed characterising the subsoil in terms of the frequency range liable to amplifying effects in the event of an earthquake. A broadband frequency peak between 1 and 10 Hz was identified which, although not characterised by significant H/V amplitude values, clearly indicates the possibility that these frequencies may be amplified during a seismic event.

Two 3D subsoil models were obtained from active and passive seismic surveys, respectively. The first shows the distribution of the longitudinal wave velocity (Vp) value, allowing the identification of low-velocity zones probably associated with cavities filled entirely or in part by backfill materials, and high-velocity area attributable to calcarenite belonging to the Ragusa formation.

The 3D model obtained by the passive seismic survey shows the distribution of the impedance contrasts, highlighting even in this case the presence of transition zones between materials with different physicomechanical features.

The quantitative evaluation of the subsoil data obtained comparing 3D models, has allowed to highlight areas where both surveys detect the presence of anomalies, thus reducing the uncertainty associated with their detection. The anomalies detected can be attributed, with good probability, to the remains of the ancient Byzantine church of San Nicola.

A cost-effective expedited ambient vibration test is operated in order to extrapolate a rapid structural characterization of the church. The dynamic identification is focused on the façade and the dome due their high vulnerability. The following modal frequencies are estimated through the SSR method: 2.34 Hz and 3.34 Hz for the first two modes of the façade, and 1.86 Hz and 1.90 Hz for the first two modes of the dome. With the aim of exploiting the advantage of using a single device for the characterisation of the whole site, a correlation between frequencies of the building with the ground ones is carried out. The former are inside the range of soil interest, thus the possibility of resonance phenomena cannot be ruled out. It is clear that this result highlights the need for further, more in-depth investigations, especially regarding the structural section. To this end, a campaign exclusively dedicated to the structural identification, on the basis of the important preliminary information obtained, will be carried out, extending the monitored parts of the structure to the right-side aisle, which is the only one accessible, and adding measurement points on the dome, in order to acquire synchronised recordings as in the façade. The envisaged results will be essential for the seismic vulnerability assessment of the building through finite element modelling.

## Figures and Tables

**Table 1:** Modal frequencies of the façade separated according to longitudinal and transversal components, comparison between SSR and FDD estimations.

Estimator	*f_SSR_*	*f_FDD_*	Δ*f*
Longitudinal Component (N-S)	2.34 Hz	2.25 Hz	4.0%
Transversal Component (E-W)	3.34 Hz	3.25 Hz	2.4%

**Table 2:** Natural periods of the dome and the façade separated according to longitudinal and transversal component. Values inside the maximum spectral acceleration range are underlined.

Macro Element	Longitudinal Component (N-S)	Transversal Component (E-W)
Dome	0.52 s	0.54 s
		0.39 s
Façade	0.42 s	0.30 s

## Data Availability

The data presented in this study are available on request from the corresponding author.
